# Unraveling genetic causality between type 2 diabetes and pulmonary tuberculosis on the basis of Mendelian randomization

**DOI:** 10.1186/s13098-023-01213-8

**Published:** 2023-11-10

**Authors:** Shengnan Chen, Weisong Zhang, Zhenquan Zheng, Xiaolong Shao, Peng Yang, Xiaobin Yang, Kai Nan

**Affiliations:** 1 Department of Joint Surgery, HongHui Hospital, Xi’an Jiaotong University, Xi’an, 710054 Shaanxi People’s Republic of China; 2 Medical Department of Xi’an Jiaotong University, Xi’an, 710048 Shaanxi China; 3 Hongdong County Hospital of Traditional Chinese Medicine, Hongdong, 041600 Shaanxi China

**Keywords:** Type 2 diabetes mellitus, Pulmonary tuberculosis, Mendelian randomization study

## Abstract

**Background:**

The comorbidity rate between type 2 diabetes mellitus (T2DM) and pulmonary tuberculosis (PTB) is high and imposes enormous strains on healthcare systems. However, whether T2DM is causally associated with PTB is unknown owing to limited evidence from prospective studies. Consequently, the present study aimed to clarify the genetic causality between T2DM and PTB on the basis of Mendelian randomization (MR) analysis.

**Methods:**

Genetic variants for T2DM and PTB were obtained from the IEU OpenGWAS project. The inverse variance weighted method was used as the main statistical analysis method and was supplemented with MR-Egger, weighted median, simple mode, and weighted mode methods. Heterogeneity was analyzed using Cochran’s Q statistic. Horizontal pleiotropy was assessed using the MR-PRESSO global test and MR-Egger regression. Robustness of the results was verified using the leave-one-out method.

**Results:**

A total of 152 independent single-nucleotide polymorphisms (SNPs) were selected as instrumental variables (IVs) to assess the genetic causality between T2DM and PTB. Patients with T2DM had a higher risk of PTB at the genetic level (odds ratio (OR) for MR-Egger was 1.550, OR for weighted median was 1.540, OR for inverse variance weighted was 1.191, OR for simple mode was 1.629, OR for weighted mode was 1.529). There was no horizontal pleiotropy or heterogeneity among IVs. The results were stable when removing the SNPs one by one.

**Conclusions:**

This is the first comprehensive MR analysis that revealed the genetic causality between T2DM and PTB in the East Asian population. The study provides convincing evidence that individuals with T2DM have a higher risk of developing PTB at the genetic level. This offers a significant basis for joint management of concurrent T2DM and PTB in clinical practice.

**Supplementary Information:**

The online version contains supplementary material available at 10.1186/s13098-023-01213-8.

## Background

Diabetes mellitus (DM) is a chronic systemic disease characterized by metabolic and endocrine disorders. DM has reached epidemic proportions with a high and increasing prevalence [1, 2]. The Global Burden of Disease Study 2021 indicated that 529 million people worldwide are living with DM, imposing considerable economic and health burdens on individuals and society [3]. DM prevalence rates are driven almost entirely by type 2 diabetes mellitus (T2DM), which accounts for 96.0% (95% uncertainty interval: 95.1–96.8) of DM cases [3]. The available epidemiological and clinical evidence supports that there is a high comorbidity rate between T2DM and pulmonary tuberculosis (PTB) [4, 5]. PTB is a chronic disease caused by *Mycobacterium tuberculosis*. According to the Global Tuberculosis Report 2022, approximately 10.6 million people fell ill with tuberculosis in 2021, a 4.5% increase compared to 2020 [6]. Similarly, the tuberculosis incidence rate is estimated to have increased by 3.6% between 2020 and 2021 [6]. Conventional wisdom holds that tuberculosis is a disease of poverty [7]. However, it is worth noting that, the incidence of tuberculosis has increased with economic and societal development. Hence, additional insights are required to account for this paradox.

Epidemiologic data suggest that individuals with diabetes are at a significantly higher risk of PTB. Animal experiments also showed that diabetic mice have increased susceptibility to PTB [8]. A systematic review of 13 observational studies reported that diabetic patients were 3.11 times (95% CI: 2.27–4.26) more likely to have tuberculosis than controls [9]. However, associations derived from observational studies are susceptible to confounders and reverse causation bias [10]. A retrospective cohort of 42,116 clients aged 65 or more years in 18 Elderly Health Service centers in Hong Kong showed that the risk of PTB is 1.89-fold higher in diabetic patients than in non-diabetic individuals [11]. Another prospective cohort study involving 17,715 Taiwanese persons observed that diabetic individuals have a 2.09-fold increase in risk for tuberculosis [12]. However, determining an estimated risk of PTB associated with T2DM was impossible because these studies did not distinguish between T1DM and T2DM. Therefore, whether T2DM is causally associated with PTB risk remains unknown. Further research is required to support the causal association between T2DM and PTB.

The genetic characterization of diseases provides a basis for disease prevention and treatment [13]. It has been proved that genetic elements such as single-nucleotide polymorphisms (SNPs) participate in the pathogenesis of T2DM and PTB [14, 15]. Multiple genome-wide association studies (GWAS) have been conducted in recent years, providing strong data to support investigation of causal associations using Mendelian randomization (MR) design [16]. MR is an epidemiological method that uses genetic variants, especially SNPs, as instrumental variables (IVs) to determine the genetic causality between an exposure and outcome [17]. The application of genetic variables minimizes the influence of reverse causality and confounding factors because genetic variants are randomly allocated at conception and cannot be modified by the development of disease [18].

Therefore, the present study aimed to investigate the genetic causality between T2DM and PTB based on the MR method to fill in the above scientific gaps. To the best of our knowledge, no prior studies have inferred a potential causal relationship between T2DM and PTB using MR analysis. These findings may provide a novel perspective for understanding the causal effect of the endocrine system on the immune system. In particular, it may offer a significant basis for the joint management of concurrent T2DM and PTB.

## Methods

### Study design

This study was a two-sample MR analysis based on publicly available GWAS data. MR analysis requires meeting three core assumptions [19]. First, IVs should be strongly associated with exposure. Second, IVs should be independent of confounders in the association between exposure and outcome. Third, IVs should only influence the outcome through exposure and not through other pathways. In the present study, T2DM was considered as the exposure, PTB was the outcome, and SNPs significantly associated with T2DM were used as IVs.

**Fig. 1:**
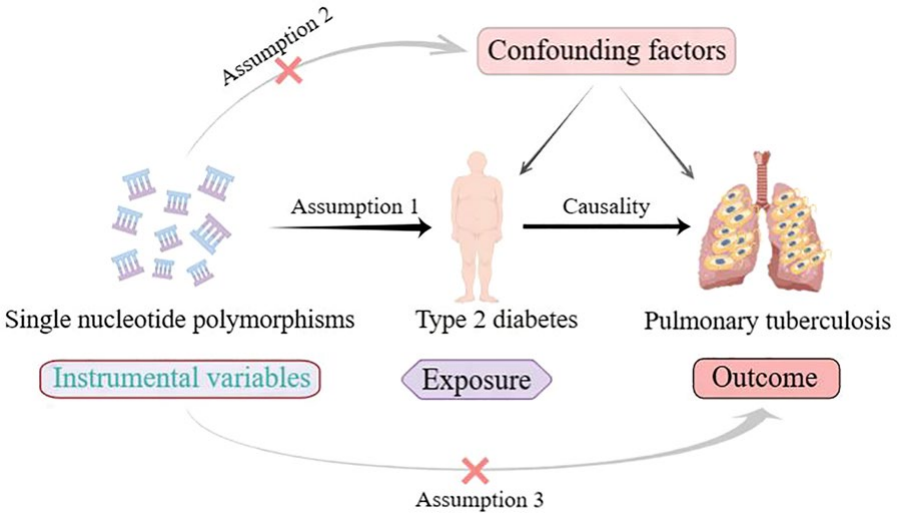
A Mendelian randomization study revealing causality between type 2 diabetes and pulmonary tuberculosis

No additional ethical approval was required because the data were retrieved from public databases. The study design is presented in Fig. 1.

### Data source

Genetic variants for T2DM and PTB were obtained from the IEU OpenGWAS project [20]. The GWAS ID for T2DM is ebi-a-GCST010118, as designated in the National Human Genome Research Institute and European Bioinformatics Institute’s (NHGRI-EBI) GWAS catalog [21, 22]. A total of 433,540 participants (356,122 controls and 77,418 cases) and 11,222,507 SNPs are included in this dataset. Data for PTB were obtained from bbj-a-149 in the BioBank Japan Project (BBJ), with a sample size of 212,453 individuals (mean age, 61.96 years; standard deviation, 13.74), including 211,904 controls, 549 cases, and 8,885,805 SNPs [23]. The population in the above datasets was the East Asian population, including males and females.

### IVs selection

SNPs without linkage disequilibrium (r^2^ < 0.001 and clump distance > 10,000 kb) were selected as IVs if they were associated with exposure at the genome-wide significance level (*P* < 5 × 10^–8^) [24].

### Statistical analysis

The inverse variance weighted (IVW) method was used as the main statistical analysis method and was supplemented by the MR-Egger, weighted median, simple mode, and weighted mode methods. The odds ratio (OR) and 95% confidence interval (CI) value was calculated accordingly. A *P-*value < 0.05 was considered statistically significant. Heterogeneity of SNPs was assessed using Cochran’s Q statistic [25]. A *P-*value > 0.05 indicated absence of heterogeneity. Horizontal pleiotropy was assessed using the MR pleiotropy residual sum and outlier (MR-PRESSO) test and the intercept obtained from the MR-Egger regression [26]. A *P-*value > 0.05 indicated absence of horizontal pleiotropy. The sensitivity analysis was performed to verify the robustness of results by using the leave-one-out method [27]. All tests were two-sided and performed using the R package TwoSampleMR version 0.5.7 in R software 4.3.1.

## Results

### Acquisition of IVs

**Fig. 2:**
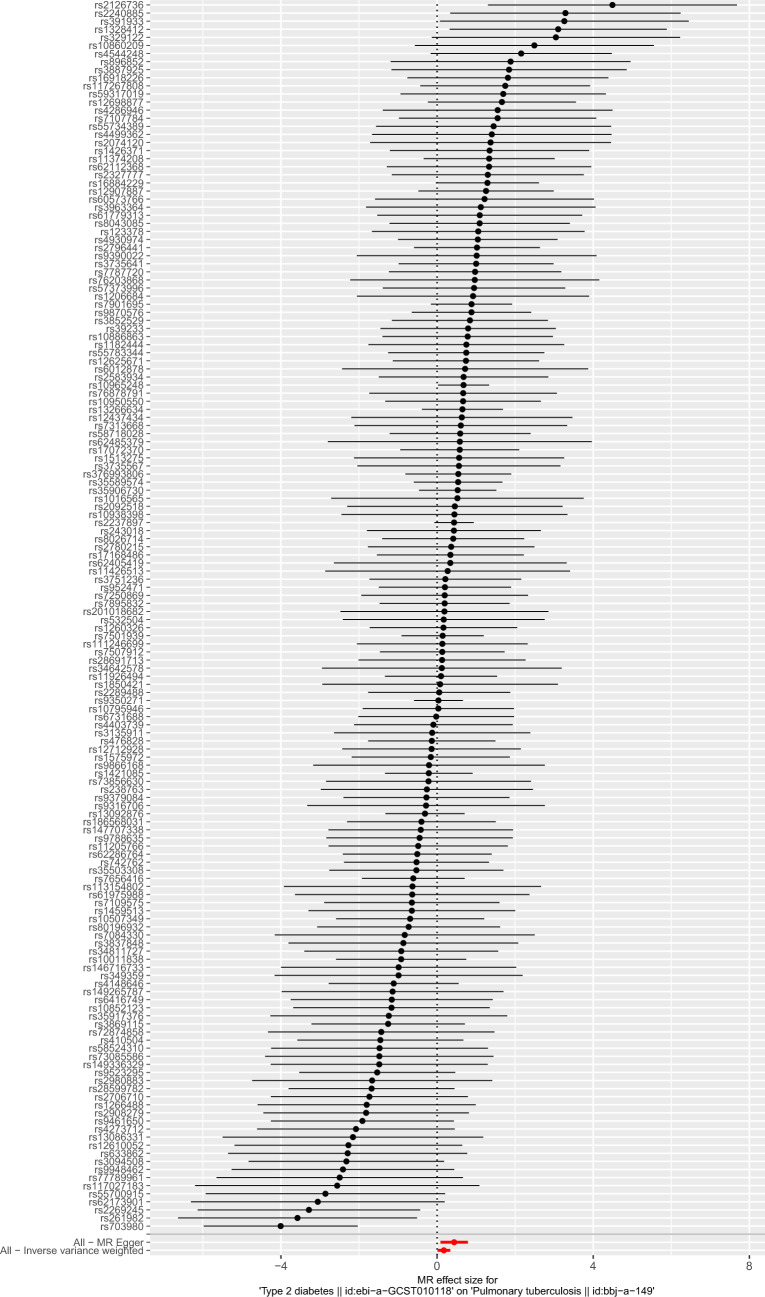
Forest plot for the effect of each SNP on pulmonary tuberculosis. The black line represents the effect produced by a single SNP, and the red line shows the causal estimate using all instrumental variables. If the solid line is completely to the left of 0, the result estimated by this SNP is that type 2 diabetes can reduce the risk of pulmonary tuberculosis. If the solid line is completely to the right of 0, the result estimated by this SNP is that type 2 diabetes can increase the risk of pulmonary tuberculosis. The result is not significant if the solid line crosses 0

Based on the above screening criteria, 174 SNPs were identified to be significantly associated with T2DM (Additional file 1: Table S1). Then, 168 SNPs out of the above 174 SNPs were extracted from PTB GWAS (Additional file 2: Table S2). The SNPs rs10830963, rs11257657, rs256904, rs340875, rs4237150, rs4736999, rs4739515, rs475002, rs6885132, rs7090695, rs7210161, rs7900112, rs8037894, rs838720, rs9368194, and rs988748 were removed for being palindromic with intermediate allele frequencies. Finally, 152 independent SNPs were selected as IVs to assess the genetic association between T2DM and PTB (Additional file 3: Table S3). The effect of each SNP on PTB is displayed in Fig. 2.

### Causal relationship between T2DM and PTB

**Fig. 3:**
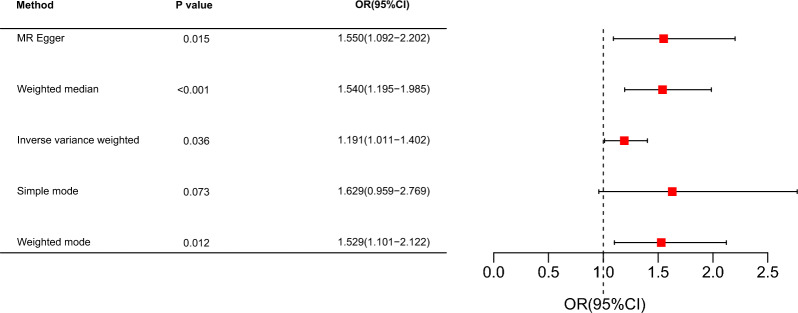
Forest plot for the effect of type 2 diabetes on pulmonary tuberculosis

**Fig. 4:**
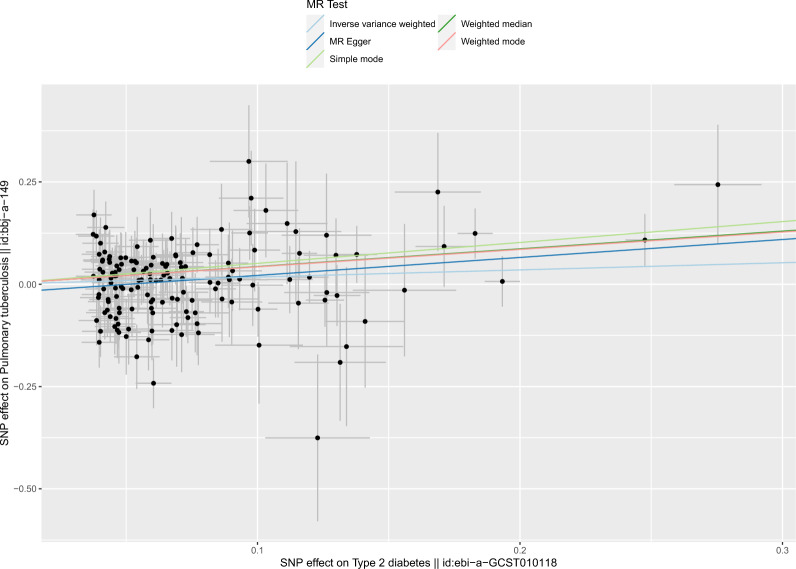
Scatter plot for the effect of type 2 diabetes on pulmonary tuberculosis. The black points represent instrumental variables. The horizontal axis represents the effect of SNPs on exposure (type 2 diabetes). The vertical axis represents the effect of SNPs on the outcome (pulmonary tuberculosis). Colored lines represent the results of MR analysis based on five methods

As shown in the forest plot (Fig. 3), patients with T2DM had a higher risk of PTB. The scatter plot (Fig. 4) also showed an increased risk of PTB in patients with T2DM.

### Sensitivity analysis

**Fig. 5:**
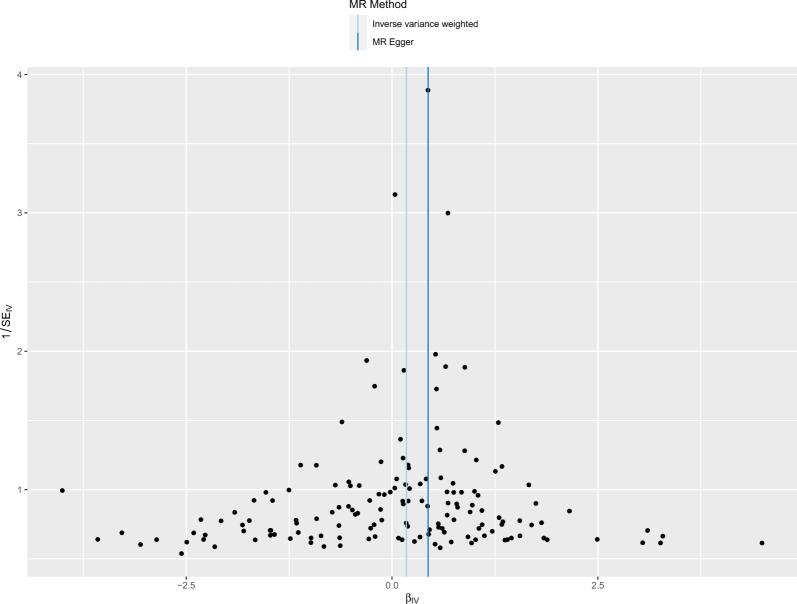
The overall heterogeneity test of the effect of type 2 diabetes on pulmonary tuberculosis. Black points represent SNPs, and the distribution of points is symmetric about the inverse variance weighted and MR-Egger line

**Fig. 6:**
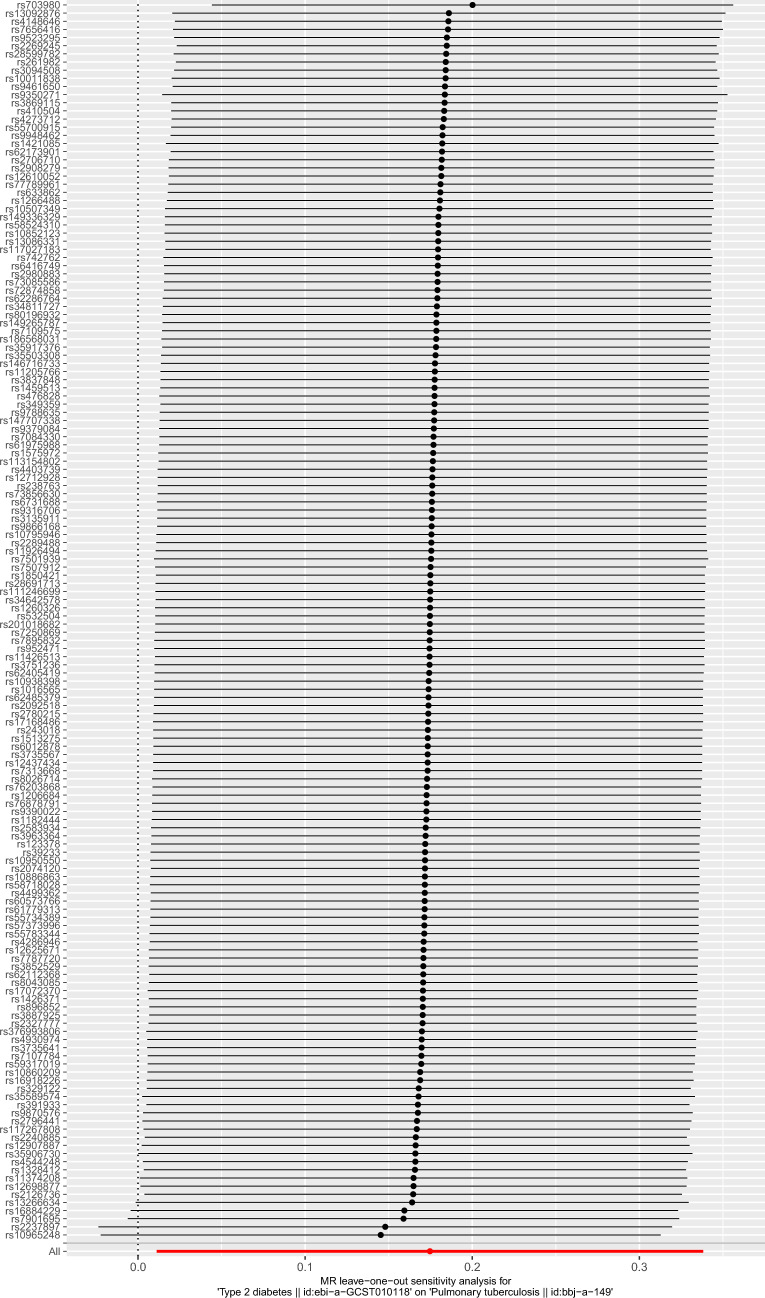
Forest plot for leave-one-out analysis. The position of the red point is greater than zero. The black dots are positioned on the right side of the invalid line. This indicates that removing any of the SNPs will not significantly impact the results

Cochran’s Q test results suggested that there was no heterogeneity among IVs (Table 1). The symmetry of the funnel plot also confirmed the absence of heterogeneity (Fig. 5). The MR-Egger intercept test and the MR-PRESSO global test, which excluded outlier variants, showed the absence of horizontal pleiotropy (Table 2). Sensitivity analysis using the leave-one-out method showed that the results were stable when removing the SNPs one by one (Fig. 6).

**Table 1:** Heterogeneity test of MR

Method	Q value	*P* value
MR-Egger Cochran’s Q test	168.0437	0.149
IVW Cochran’s Q test	171.1424	0.125

**Table 2:** Pleiotropy test of MR

Method	Value	*P* value
Intercept in MR-Egger regression	− 0.022	0.098
RSSobs in MR-PRESSO global test	173.279	0.124

## Discussion

Our comprehensive MR analysis found that T2DM, characterized by a genetic predisposition, has a genetically causal effect on PTB in the East Asian population. We conducted several sensitivity analyses to ensure the robustness of our findings, including the MR-PRESSO global test, the MR-Egger intercept test, and Cochran’s Q test. The results of the Cochran’s Q test showed no heterogeneity among the IVs. Both the MR-PRESSO global test and the MR-Egger intercept test provided evidence for the absence of horizontal pleiotropy. This suggests that IVs are unlikely to affect PTB risk through pathways other thanT2DM. The IVW method provides robust and unbiased estimates in the absence of directional pleiotropy and heterogeneity. Meanwhile, beta values for all methods were in the same direction. Leave-one-out analysis and funnel plots showed that the estimates were not biased by a single SNP, further proving the robustness of our results. Therefore, from the perspective of MR analysis, we can conclude that T2DM and PTB have a genetically causal relationship. Individuals with T2DM have a higher risk of PTB at the genetic level (OR for IVW was 1.191). Therefore, common epigenetic markers and pharmacological targets for T2DM and PTB may be useful for precise and optimal therapy for patients with concurrent T2DM and PTB. Meanwhile, the superimposition of a typical chronic non-communicable disease and an infectious disease undoubtedly provides a deeper understanding of the association between the endocrine and immune systems.

Endocrine aberrancies may contribute to changes in immune responses [28]. Previous studies have suggested that a weakened immune system in diabetic mice predisposes to *Mycobacterium tuberculosis* infection [29]. Given the high mortality rate in patients with PTB and comorbid T2DM, the control of T2DM and PTB remains a great challenge. In our study, patients with T2DM had a 1.19-fold higher risk of developing PTB at the genetic level, which is lower than that observed in previous investigations. This difference can be attributed to two factors. On the one hand, observational studies can prove correlation but not causality relationship [30]. ORs obtained from observational studies may overestimate the causal relationship between diabetes and tuberculosis. On the other hand, factors other than genetic variables may also affect the risk of PTB in patients with T2DM in real-world research.

Considering the clinical burden and causal relationship of T2DM and PTB, the joint management of T2DM and PTB should be emphasized. A previous study implicated glycine level in the evolution of PTB in patients with T2DM, indicating that nutritional status is important in the development of PTB in T2DM [31]. Diet is a significant factor affecting nutritional status [32]. However, contradictions exist in the dietary recommendations for PTB and T2DM. Patients with PTB must strengthen nutrition without restricting caloric intake. On the contrary, patients with T2DM should control blood glucose by limiting carbohydrate intake. Therefore, actions to address diabetes and undernutrition are already incorporated into the global tuberculosis strategy [33]. A study involving 1,661 East Asian participants showed that a balanced diet rich in high-quality protein, sufficient energy, and marine n-3 polyunsaturated fatty acids, phytochemicals, vitamin B, and fiber is associated with mild clinical manifestations in patients with tuberculosis, especially those with comorbid diabetes [32]. Another study demonstrated that antidiabetic agents such as metformin and sulfonylureas are protective against long-term all-cause death in patients with concurrent T2DM and PTB [4]. Thus, metformin has been recognized as the first-line agent for reducing glucose levels and as an adjunct to anti-tuberculosis therapy in diabetic patients with tuberculosis [34]. This may be because that higher glucose concentrations are more favorable for the growth of *Mycobacterium tuberculosis*. Therefore, in addition to genetic factors, the balance between nutritional and diabetic status contributes to the prevention and control of PTB.

The present study has the following advantages compared with previous investigations. To the best of our knowledge, this is the first MR study that clarified the genetically causal relationship between T2DM and PTB in the East Asian population. The major strength of the present study is the MR design, which minimized the influence of reverse causality and bias from confounding factors. Second, samples from GWAS data in the present study are large enough to estimate the causal relationship between T2DM and PTB. Third, the populations represented by the two GWAS datasets were both East Asian, which effectively avoids racial differences. Fourth, five methods were carried out to determine the causal relationship between T2DM and PTB, which increased the credibility of the findings.

This study also exhibits some limitations. First, the results were derived from the East Asian population. Hence, their generalizability to other races may be limited. Second, background factors such as age, sex, and duration of disease could not be obtained and analyzed as stratification factors. Therefore, studies across a range of populations and ethnic groups employing large sample size and various stratification factors are required to further prove the present findings.

## Conclusions

This is the first comprehensive MR analysis that revealed the genetically causal relationship between T2DM and PTB in the East Asian population. The study provides convincing evidence that individuals with T2DM have a higher risk of PTB at the genetic level. These findings provide a novel perspective for understanding the causal effect of the endocrine system on the immune system. In particular, they offer a significant basis for the joint management of concurrent T2DM and PTB in clinical practice. Further studies are needed to identify common epigenetic markers and pharmacological targets for precise and optimal therapy for patients with T2DM-PTB comorbidity.

### Supplementary Information

**Additional file 1: Table S1. **SNPs significantly associated with T2DM at the genome-wide level.

**Additional file 2: Table S2. ** Identified SNPs in PTB GWAS.

**Additional file 3: Table S3. **IVs used to assess the genetic association between T2DM and PTB.

## Data Availability

The datasets analyzed during the current study are available in the IEU OpenGWAS project.

## Abbreviations

DM: Diabetes mellitus
T2DM: Type 2 diabetes mellitus
PTB: Pulmonary tuberculosis
SNPs: Single-nucleotide polymorphisms
GWAS: Genome-wide association study
MR: Mendelian randomization
IVs: Instrumental variables
IVW: Inverse variance weighted
OR: Odds ratio
CI: Confidence interval
MR-PRESSO: MR pleiotropy residual sum and outlier
